# High incidence and poor prognosis of bone metastases in functioning small intestinal neuroendocrine tumors

**DOI:** 10.3389/fendo.2025.1680209

**Published:** 2025-10-23

**Authors:** Annie Mathew, Pia Hauptmeier, Benedikt M. Schaarschmidt, Wolfgang P. Fendler, Dagmar Führer, Harald Lahner

**Affiliations:** ^1^ Department of Endocrinology, Diabetes and Metabolism and Division of Laboratory Research, University Hospital Essen, University of Duisburg-Essen, Essen, Germany; ^2^ Endocrine Tumor Center at Westdeutsches Tumorzentrum (WTZ)/Comprehensive Cancer Center and European Neuroendocrine Tumor Society (ENETS) Center of Excellence, University Hospital Essen, University of Duisburg-Essen, Essen, Germany; ^3^ Institute for Diagnostics and Interventional Radiology and Neuroradiology, University Hospital Essen, University of Duisburg-Essen, Essen, Germany; ^4^ Department of Nuclear Medicine, German Cancer Consortium [Deutsches Konsortium für Translationale Krebsforschung (DKTK)], University Hospital Essen, University of Duisburg-Essen, Essen, Germany

**Keywords:** bone metastases, neuroendocrine tumors, small intestine, ileum NET, functioning NET, DOTATOC-PET/CT

## Abstract

**Background:**

The prevalence and clinical relevance of bone metastases (BM) in advanced small intestinal neuroendocrine tumors (siNETs) is not well-documented.

**Methods:**

We analyzed data from 458 patients (54% male, median age 58 years) with histologically confirmed siNETs treated at the ENETS Center of Excellence Essen from 2003 to 2023. BM occurrence and their impact on skeletal-related events (SREs) and overall survival (OS) were assessed using standardized DOTATOC-PET/CT within a consistent “one-stop shop” multidisciplinary care model.

**Results:**

At diagnosis, 305/458 patients (66.6%) had stage IV disease; BM were detected in 105/305 (34.4%). Functioning tumors were more frequent in BM patients (73%) than in the total cohort (40%). In 48.6% of patients, BM were initially visible on SSTR imaging only, becoming morphologically detectable after a median of 16 months. Most BM were osteoblastic (58%). During a median follow-up of 36 months, SREs occurred in 12.4% of BM patients, predominantly in those with osteolytic disease. SREs occurred in 27% of patients without antiresorptive therapy, but in none with treatment (p < 0.0001). Median OS was significantly shorter in patients with BM (127 vs. 170 months, p = 0.023), independent of age, sex or tumor grade.

**Conclusion:**

BM are frequent in siNET, particularly in functioning tumors, and are associated with reduced survival. BM may initially be detectable only by functional imaging but becomes morphologically visible within less than 1.5 years. Antiresorptive therapy may reduce SREs. Whether adapting NET treatment algorithm for BM improves OS needs to be tested in clinical trials.

## Introduction

Neuroendocrine tumors of the small intestine (siNETs) are rare neoplasms with a rising annual incidence of 1.2 per 100,000 (1). At diagnosis the majority of patients present with locally advanced or metastasized disease requiring palliative management (2). Prognosis and therapeutic concepts are mainly based on grading and staging and well as localization of NET according to the 2022 World Health Organization (WHO) classification (3). Other factors influencing survival remain poorly understood so far. In addition, systemic treatment options for advanced disease have expanded in recent years. Alongside somatostatin analogues, targeted agents and peptide receptor radionuclide therapy, chemotherapy continues to play a relevant role, particularly in pancreatic NETs and in patients with high-grade or rapidly progressive disease, as highlighted in recent reviews (4, 5).

Small intestine NETs constitute the most prevalent gastrointestinal NETs and potentially metastasize to any organ, with the liver being the most commonly affected (6). Diagnosis of bone metastases (BM) in NET depends on the detection method by SSTR scintigraphy, CT, MRI, bone scintigraphy or hybrid functional imaging (7). Accordingly, previous studies reported variable BM rates from 5.7% to 26% in NET cohorts (8), yet lack standardization in terms of tumor characteristics (e.g., NET location, grading, staging), applied imaging methods and are not informative on clinical courses (9, 10).

In a previous study, we showed that BM occurred with considerably higher frequency than previously thought, are present in more than one-third of advanced PanNETs (36,3%) and are associated with reduced survival based on systematic use of 68Ga-DOTATOC PET/CT (11). In our present study, we likewise applied 68Ga-DOTATOC-PET/CT as the gold standard for NET imaging and addressed the prevalence and prognostic impact of BM in NET patients at our ENETS center in which all patients are under long-term “one-stop shop care” by the same expert team and where disease course and therapeutic strategies are regularly reviewed in a multidisciplinary tumor board.

## Materials and methods

**Figure 1:**
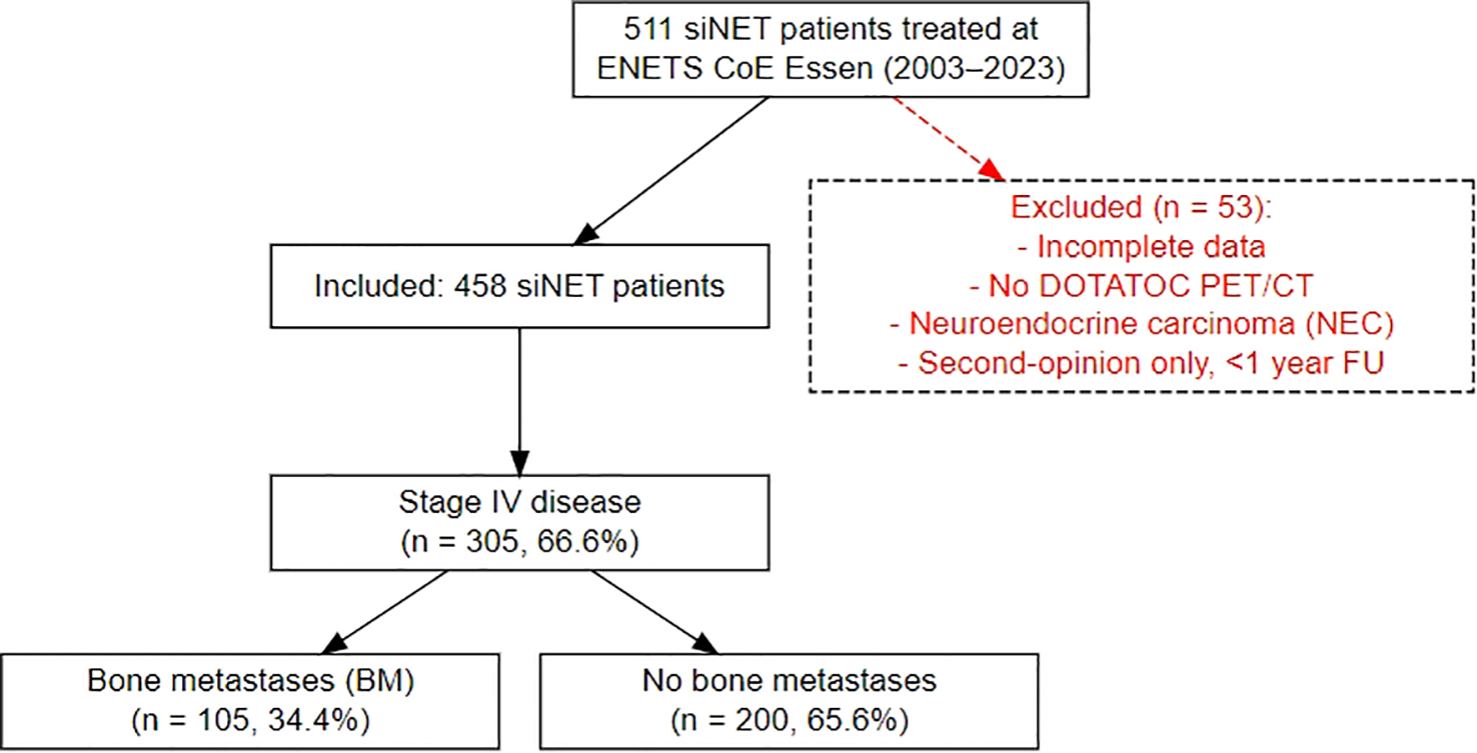
Flowchart of patient selection and study cohorts.

Patients were identified from our prospective NET database at the European Neuroendocrine Tumor Society (ENETS) Center of Excellence (CoE), Department of Endocrinology, Diabetes and Metabolism, University Hospital Essen. Eligible patients included those with histologically confirmed differentiated siNETs who were treated at our department between February 2003 and May 2023 (**Figure 1**). All patients underwent contrast-enhanced 68Ga-DOTATOC-PET/CT at initial presentation and at subsequent follow-up. Patients with incomplete data were excluded from further analysis. To ensure consistency, scheduling of visits as well as indications for therapies was determined according to ENETS guidelines by an experienced, multidisciplinary tumor board, comprising board-certified endocrinologists, pathologists, surgeons, oncologists, radiologists and nuclear medicine specialists. All staging was performed in-house at our ENETS center.

The presence of BM was documented as a) BM by morphological evidence, b) BM by pathological tracer uptake only or c) BM by morphological evidence and pathological tracer uptake.

Skeletal-related events (SREs) were defined as the presence of pathological fractures, bone surgery, bone radiation and/or metastatic spinal cord compression. Antiresorptive treatment was recommended to all patients at initial diagnosis of BM and included either monthly zoledronate (4 mg) or denosumab (120 mg).

Categorical variables were reported as counts and percentages; continuous data as medians with 95% confidence intervals. Overall survival (OS) was estimated using the Kaplan-Meier method and compared with the results from the log-rank test. The tests were two-tailed and results at p < 0.05 were interpreted as statistically significant. All statistical analyses were performed using the Statistical Package for the Social Sciences version 26.0 software program (IBM Corporation, Armonk, NY, USA).

The study was approved by the Ethics Committee of the Medical Faculty of the University of Duisburg-Essen (18-8367-BO).

## Results

A comprehensive analysis was conducted on the clinical records of 458 consecutive patients diagnosed with histologically confirmed siNETs (siNET cohort) at the ENETS Excellence Center in Essen from 2003 to 2023. The data was extracted retrospectively from the Essen NET database, which contains clinical characteristics, histology, endocrine and biochemical parameters, results of advanced imaging, treatment lines and outcome, of all NET patients treated and followed up in a “one-stop shop care” principle. Of 458 patients (249 male, median age of 58 years (range 19-84) at NET diagnosis), 305 showed distant metastases, representing advanced disease (stage IV siNET cohort). Within this subset and during a median follow-up of 36 months, 105 patients (34.4%) presented or developed BM (BM siNET cohort) identified by increased radiotracer uptake and/or morphological criteria with more men (61.9%) than women affected. Details outlining the distinct characteristics of the three Essen siNET cohorts are shown in **Table 1**.

**Table 1:** Comparison of the siNET cohort, stage IV siNET cohort and BM siNET cohort at ENETS CoE Essen, p-values refer to comparisons between the stage IV siNET and BM siNET cohort.

Parameter	Essen siNET cohort	Essen stage IV siNET cohort	Essen BM siNET cohort
Patient characteristics			
Total patients	458 (100%)	305/458 (66.6%)	105/305 (34.4%)
Median age at diagnosis	58 years (range 19-84)	59 years (range 19-84)	60 years (range 19-83)
Median follow-up period	39 months (range 0-240)	37 months (range 0-240)	37 months (range 0-202)
Sex			**p = 0.186**
Male	249 (54.4%)	173 (56.7%)	65 (61.9%)
Female	209 (45.6%)	132 (43.4%)	40 (38.1%)
Localization			**p = 0.402**
Ileum	73.8% (338/458)	74.8% (228/305)	73.3% (77/105)
Jejunum	7.4% (34/458)	6.6% (20/305)	6.7% (7/105)
Duodenum	6.3% (29/458)	3.9% (12/305)	1.9% (2/105)
Undetermined small intestine	12.4% (57/458)	14.8% (45/305)	18.1% (19/105)
Grading			p = 0.037*
G1	62.2% (285/458)	57.1% (174/305)	52.4% (55/105)
G2	35.6% (163/458)	39.7% (121/305)	41.9% (44/105)
G3	1.7% (8/458)	2.6% (8/305)	5.7% (6/105)
Functionality			p < 0.001***
Functioning	40% (183/458)	56.1% (171/305)	73.3% (77/105)
- Serotonin	- 97.8% (179/183)	- 98.8% (169/171)	- 98.7% (76/77)
- Gastrin	- 2.2% (4/183)	- 1.2% (2/171)	- 1.3% (1/77)
Non-Functioning	60% (275/458)	43.9% (134/305)	26.7% (28/105)

Bold p-values refer to comparisons between the stage IV siNET and BM siNET cohort.

### Relevance of imaging mode and morphology of siNET BM

**Figure 2:**
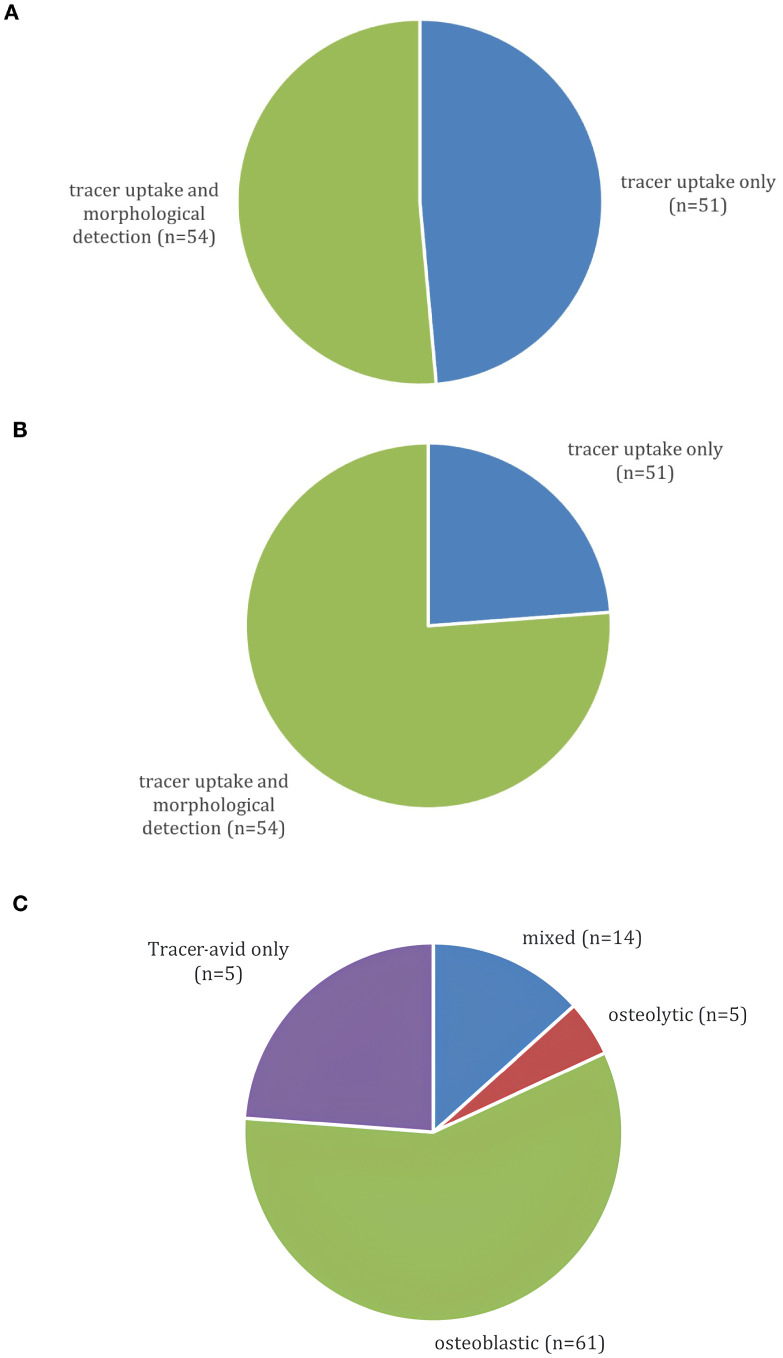
**(A)** Presentation of BM on 68Ga-DOTATOC-PET/CT at initial BM diagnosis (n=105). **(B)** Presentation of BM on last 68Ga-DOTATOC-PET/CT at data cut-off (n=105). **(C)** Morphology of BM on 68Ga-DOTATOC-PET/CT (n=105).

At initial diagnosis (**Figure 2A**), BM were detected via tracer-avid and morphologically visible metastases in 51.4% of patients, whereas 48.6% had BM visible by tracer-uptake only. These BM became morphologically apparent after a median of 16 months (range 3-198). At the time of data cut-off, tracer-avid and morphologically visible metastases were documented in 76.2% and 23.8% showed tracer-avid-only metastases (**Figure 2B**). The morphologically visible BM were further classified into osteoblastic (58%), osteolytic (4.8%) or mixed (13.3%) metastases (**Figure 2C**).

### Relevance of grading, location and functional activity

Forty-three siNET patients (41%) showed synchronous metastases within the first six months after the initial NET diagnosis and 62 patients (59%) developed BM during further follow-up. The median time from initial siNET diagnosis to first manifestation of BM was 16 months. Among the 105 patients with BM, 55 (52.4%) had a G1 tumor, 44 (41.9%) had a G2 tumor and six (5.7%) had a G3 tumor. Depending on the primary site, BM were diagnosed in 33% of ileum NET (77/228), 35% of jejunal NET (7/20), 42.2% of non-specified siNET (19/45) and in 2/12 duodenal NET. Almost all patients with BM (96,2%) presented with other distant organ metastases, predominantly to the liver (94,3%), either alone (31,4%), or in combination with metastases to peritoneum or lungs and less frequently to the heart, reproductive organs or the pancreas. Only 5.7% of patients represented with metastatic siNET where the liver was not involved. In the Essen siNET cohort, 40% (183/458) of patients had functioning tumors, with serotonin-producing tumors comprising the vast majority (97.8%). This proportion increased notably in the stage IV subgroup, where 56.1% (171/305) of patients had functioning tumors. Remarkably, 73,3% of siNETs with BM were functioning (98.7% serotonin excess) and only 26.7% were non-functioning, in contrast to the total siNET cohort comprising 40% functioning and 60% non-functioning siNET (p<0.001; odds ratio (OR) of 3.1 (95% CI 1.9-5.2).

### Skeletal distribution of BM in siNET

“Thirty-nine percent of patients exhibited multifocal skeletal involvement with four or more bone lesions across different sites. Spinal bone metastases limited to 2–3 sites were present in 31.4% of patients. Solitary BM (spine, pelvis, sternum, rib and scapula) were documented in 21% of patients.

### Skeletal-related events and therapy

Incidence of SREs such as a fracture, pain, bone-related surgeries, radiation or spinal cord compressions was documented. SREs were observed in 12.4% of BM patients with fractures (8/13), pain (5/13) and bone radiation (5/13) being the most common SREs. Spinal cord compression occurred rarely (3/13 patients, 23.1%). Start of antiresorptive therapy in the bone metastases regimen, e.g. denosumab 120 mg monthly or bisphosphonates such as zoledronate 4 mg monthly was recommended to all patients at initial diagnosis of BM. Denosumab was preferred in patients with renal insufficiency, while patient preference also played a role, particularly regarding the route of administration. Among 105 patients, 27.6% received therapy while 72.4% did not.

SREs occurred in 27.7% of patients without antiresorptive therapy within a median of 41 months (range 0–248 months) after initial siNET diagnosis and a median of 12 months (range 0–115 months) after diagnosis of BM. None of the patients presented with SREs under antiresorptive therapy during follow-up (p<0.0001). SREs were less common in BM with osteoblastic morphology (11.5%) compared to BM with mixed or osteolytic morphology (40%).

### Impact of siNET BM on overall survival

In stage-IV siNET patients, the median OS since NET diagnosis was 145 months (95% CI, 117.5-172.5). During the observation period (2003–2023), 45 patients with BM (42.9%) and 59 patients without BM (57.1%) passed away. From the first manifestation of BM to the patient’s death, a median of 29 months (95% CI, 28.4-53) elapsed.

**Figure 3:**
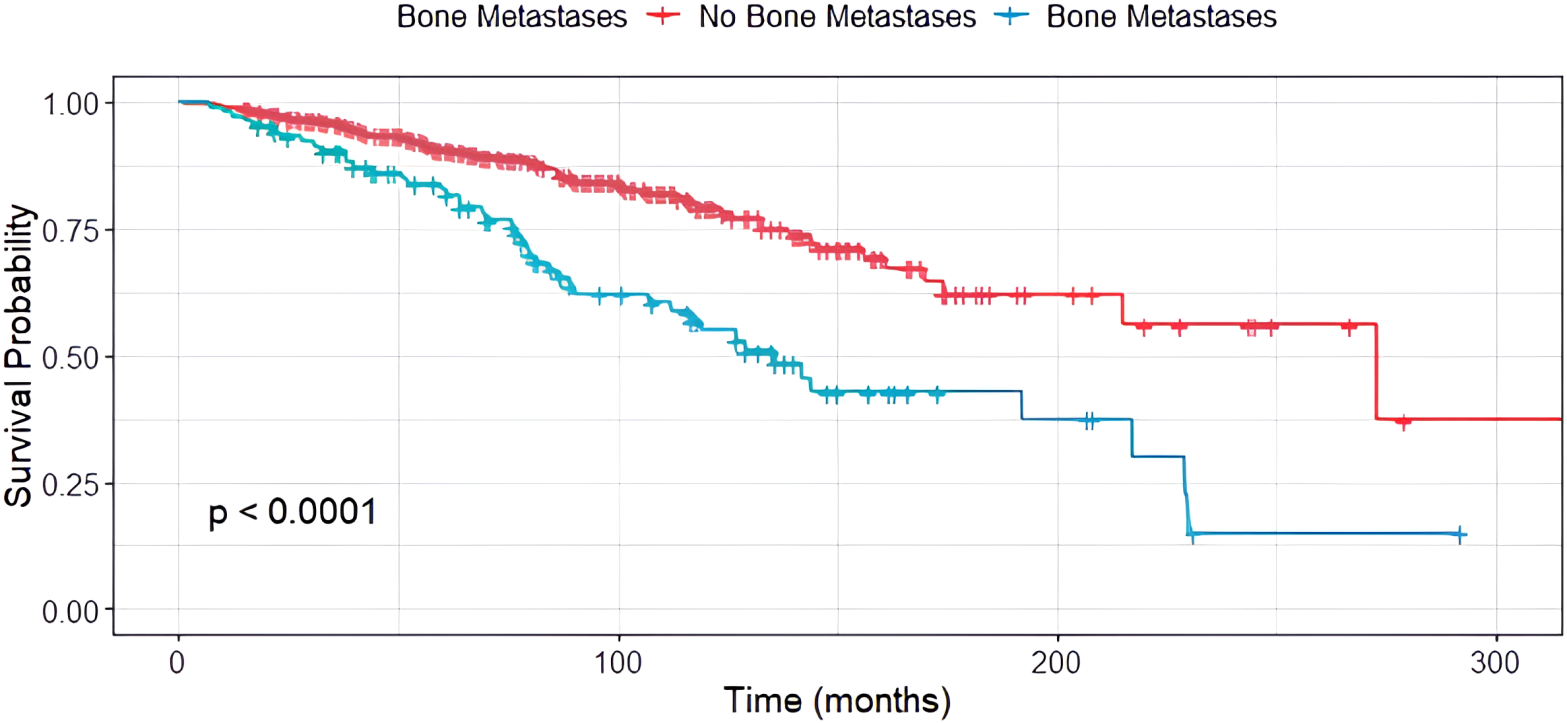
Median OS in siNET patients with distant metastases other than bone (red) and bone metastases (green), n=305.

Compared to patients without BM, the median OS in siNET with BM was substantially reduced (170 months (95% CI, 117.7–222.3) vs. 127 months (95% CI, 103.1–150.9); **Figure 3**), and the presence of BM was a stronger negative predictor than grading in G1 and G2 siNET (G1: 156 months (95% CI, 109–203); G2: 144 months (95% CI, 113.3–174.7)). Due to the smaller group of G3 NET patients, survival analysis was omitted for this patient group. In our siNET cohort, the overall 5-year survival rate was 88.8%, and the 10-year survival rate was 72.6%, based on Kaplan-Meier estimates.

When comparing siNET with different metastasizing patterns, the median OS of patients with BM was 51 months less than in patients with advanced siNET but absence of BM (119 months (90.7-147.3, 95% CI) vs. 170 months (139.5-200.5 months, 95% CI)).

Furthermore, synchronous metastases were associated with reduced survival (119 months) compared to metachronous metastases 136 months (p=0.055). Interestingly, no difference in survival was found between patients with metastases visible by tracer-uptake only compared to patients with BM visible by tracer-uptake and CT scan (119 vs 127 months, p=0.305). SREs occurred in 10.4% of patients with PET-only bone metastases and in 14.8% of those with morphologically visible lesions. This difference was not statistically significant (p = 0.56), indicating comparable clinical relevance of functionally detectable metastases.

Patients with osteoblastic BM had a median OS of 136 months, whereas those with osteolytic BM had a median OS of 77 months. The difference was not statistically significant (p = 0.3).

**Figure 4:**
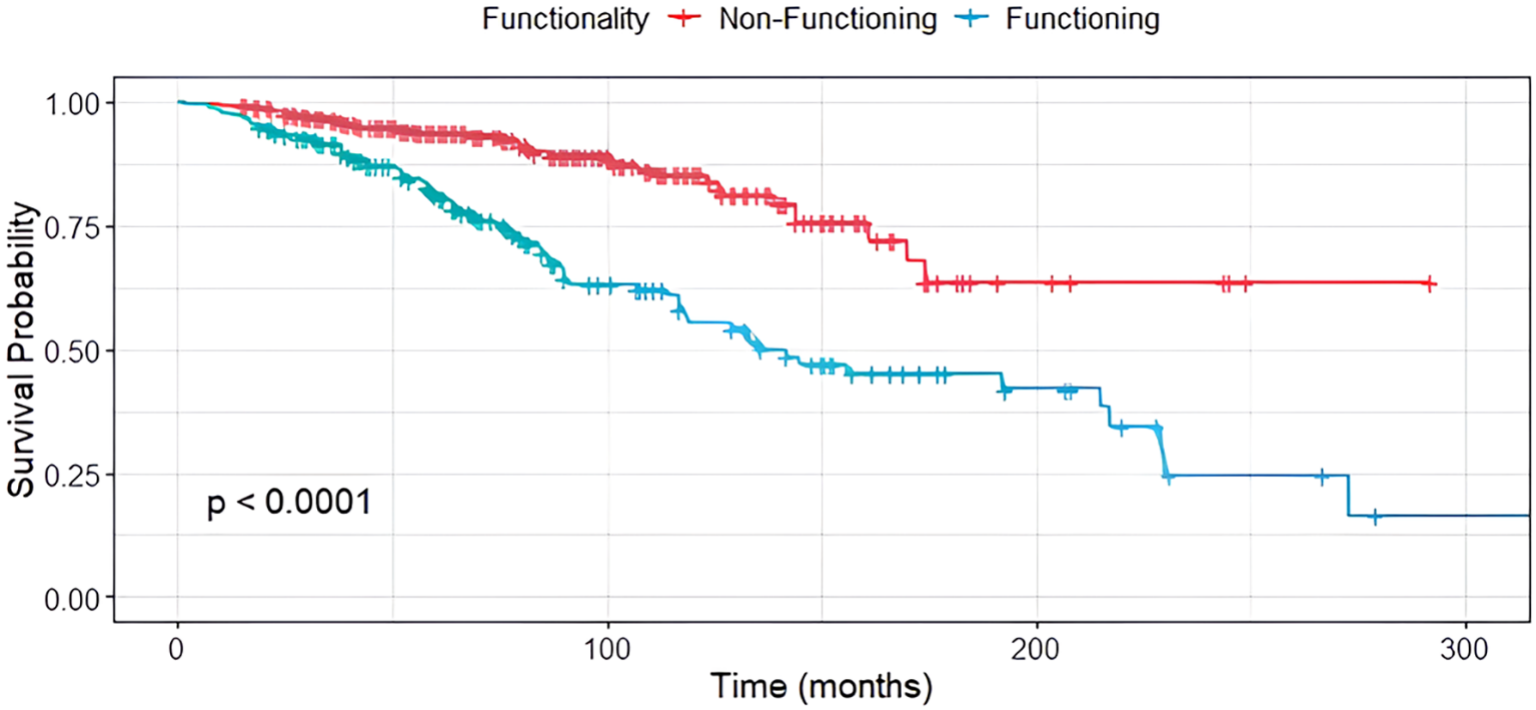
Median OS in non-functioning (red) and functioning (green) siNET, n=305.

**Figure 5:**
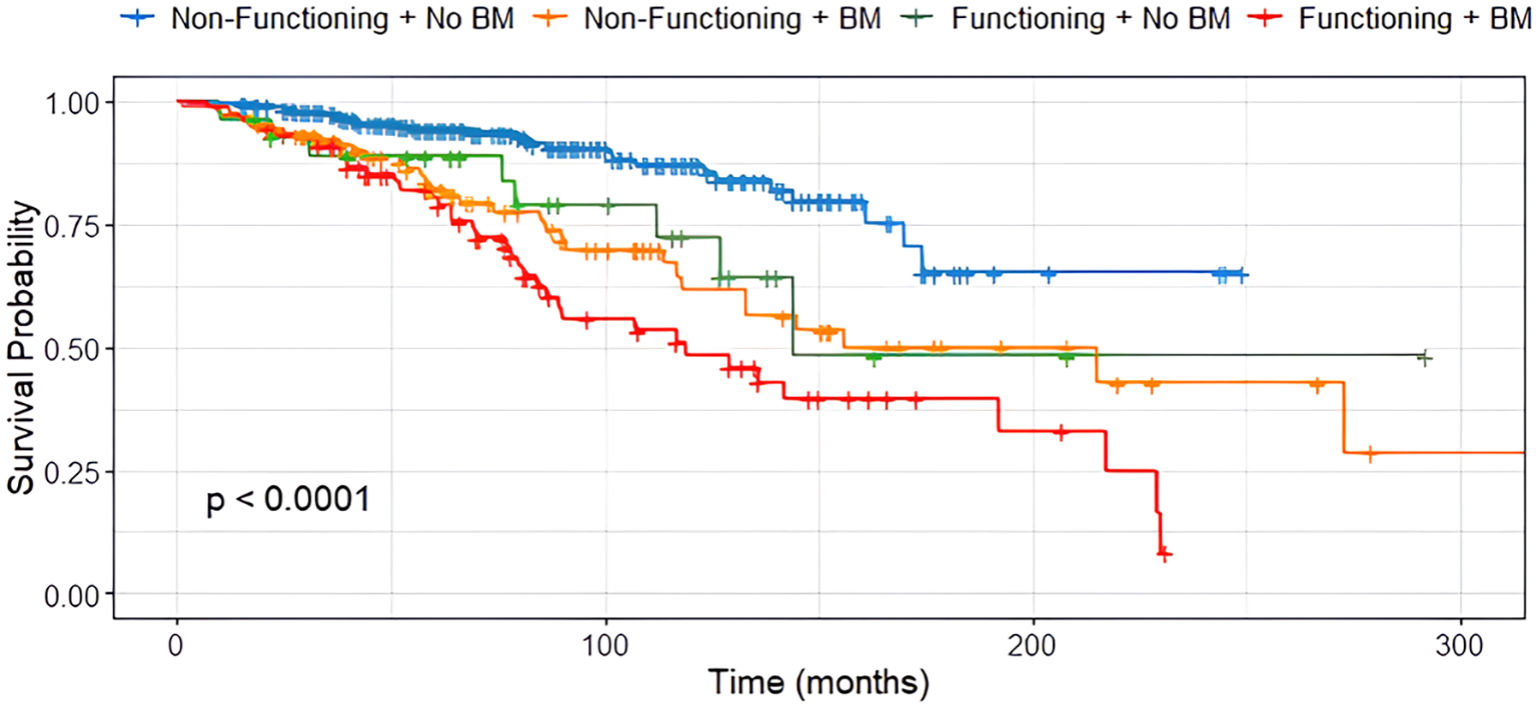
Median OS in non-functioning and functioning siNET with and without BM, n=305.

In contrast, tumor functionality was significantly associated with survival (p < 0.001), with non-functioning siNET showing notably better overall survival compared to functioning siNET, particularly in the presence of BM (**Figure 4**). The shortest OS was observed in patients with functioning tumors and BM (119 months), while patients with non-functioning tumors without bone involvement had the most favorable outcome, with the median OS not yet reached (**Figure 5**).

Survival was neither correlated to gender nor the location of the primary tumor (p=0.136).

## Discussion

Precise data on the prevalence and impact of BM in NET outcomes is still rare. Our study highlights that, using advanced imaging, more than one-third of siNET patients develop BM, mirroring previous findings from our group in panNET patients, where BM incidence reached 36.3% (11). This is the highest incidence published to date in a histologically homogeneous NET cohort, and it supports the hypothesis that BM may be significantly underdiagnosed in NETs overall when assessed using conventional imaging methods. The current study extends this observation to siNETs, where we identified a similarly high BM incidence of 34.4% among stage IV patients.

The high rate of BM in gastrointestinal NET contrasts with earlier studies by others reporting a BM incidence of only 3.6%, 5.4%, 6.4%, 11.7%, 11.9% and 26.0% in NET (1, 7, 12–15). These analyses were conducted using multicenter cohorts and retrospective databases, included NETs of various primary sites, and differed in imaging techniques applied. The cohorts included patients with GEP-NEN, CUP-NEN, and pulmonary NEN and used various imaging modalities such as MRI, DOTATOC-PET/CT, somatostatin receptor scintigraphy, CT, bone scintigraphy and X-ray.

Our study stands out due to its large patient cohort of 458 siNET patients. It specifically examines the impact of bone metastases, distinguishing between those present at diagnosis and those that develop later. With long-term follow-up data, this study provides valuable insights that many others lack.

The analysis of patient characteristics in the BM cohort revealed no significant age-based differences in the occurrence of BM. According to a Swedish registry, BM were reported less frequently in women (12). In our cohort, there was a similar trend towards a higher prevalence of BM in male (p=0.186).

### Relevance of imaging mode and morphology of siNET BM

Contrary to previous reports associating higher tumor burden with the development of BM, our study challenges this notion by suggesting that the detection of BM may have been delayed in earlier studies due to less sensitive imaging techniques. The use of advanced imaging such as DOTATOC-PET/CT likely contributed to the early identification of BM in our cohort. This discrepancy emphasizes the importance of considering advancements in imaging technology when interpreting historical data and underscores the evolving landscape of diagnostic capabilities. Advanced imaging contributed to the detection of BM at initial diagnosis where BM were apparent by tracer-uptake in 48.6% cases.

In pheochromocytoma and paraganglioma, SSTR PET/CT was shown to outperform MRI, CT and FDG PET/CT in detecting spinal bone metastases (16).

Evidence from other cancers, e.g., breast cancer, has shown that PET/MRI outperformed CT in detecting bone and lymph node metastases (17–19). Based on this 68Ga-DOTATOC-PET/MRI may enable earlier BM detection in NET patients, particularly in younger or renally impaired individuals while minimizing radiation exposure.

The morphological analysis of BM revealed a predominance of osteoblastic metastases (61.9%), consistent with prior reports using advanced imaging. Although the survival difference between patients with osteoblastic and osteolytic metastases was not statistically significant (p = 0.3), the shorter median OS in the osteolytic group (77 months vs. 136 months) may reflect a more aggressive clinical course.

The strong presence of liver metastases (96.2%) among patients with BM highlights that skeletal involvement rarely occurs in isolation and is often part of widespread metastatic disease.

### Relevance of grading, location and functional activity

In our cohort, higher tumor grade was associated with a greater proportion of BM, with G3 NETs comprising 5.7% of the BM group compared to only 1.7% in the overall siNET cohort. Although the majority of BM patients had G1 and G2 tumors, this shift toward higher grade suggests that tumor proliferation activity may also influence metastatic potential to the bone.

The majority of siNETs originated from the ileum (73.8%). This remained consistent across stage IV (74.8%) and BM subgroups (73.3%). Ileal tumors are more frequently detected due to hormone-related symptoms from serotonin-producing enterochromaffin cells, as well as complications such as obstruction or pain that lead to clinical investigation. These combined factors likely explain the consistent predominance of ileal siNETs observed in our and other cohorts (20).

The progressive increase in the proportion of functioning tumors, particularly serotonin-producing tumors, in stage IV (56.1%) and among patients with bone metastases (73.3%) suggests a potential association between tumor functionality and disease aggressiveness or metastatic potential. The high frequency of serotonin-producing NETs in the BM cohort may reflect a distinct tumor phenotype with higher metastatic capacity.

### Skeletal distribution of BM, SREs and therapy

The frequent presentation of multifocal skeletal lesions, particularly in the spine, highlights the clinical need for systematic skeletal assessment in siNET (**Table 2**). Although the SRE rate in our cohort was relatively low (12.4%), these events, most commonly pain, can have profound impacts on patient mobility and quality of life. Early identification and bone-specific therapy may therefore not only reduce complications but also improve functional outcomes in siNET patients. Antiresorptive therapy showed promise in reducing SRE risk with none of the patients experiencing SREs under antiresorptive therapy in comparison to a higher rate of 19.1% among those not receiving bone-specific therapy (p<0.0001). Meanwhile, antiresorptive therapy should be offered to all patients with BM, however, it is important to note that this finding is based on a retrospective design and does not establish causality.

**Table 2:** Localization of BM metastases in the BM siNET cohort (n=105).

Localization	Percentage	n
Multifocal whole skeleton (≥4 loci)	39%	41
Spine (≥2–3 loci)	31.40%	33
Whole skeleton (≥2–3 loci)	7.60%	8
Single metastases	21.90%	23
- Spine		11
- Pelvis		7
- Sternum		3
- Rib		1
- Scapula		1

Pathological fractures, pain and bone radiation were the most common SREs. The literature states that pain is the predominant symptom in patients with NET BM, significantly impacting the quality of life, especially in those with extended survival periods. An examination involving 85 individuals with NETs metastasizing to the bone revealed that 28% and 14% of subjects reported bone pain at the initial diagnosis and during follow-up, respectively (7). In contrast, much higher SRE rates have been reported in paraganglioma, with 67.4% of patients experiencing events, most commonly pain, fractures, and neurological complications (16). Tumor-specific nuclear medicine approaches include 223Radium-dichloride, which is approved for castration-resistant prostate cancer with bone-only disease but has no corresponding data in NET, whereas peptide receptor radionuclide therapy (177Lu-DOTATATE PRRT) is a systemic treatment option for SSTR-positive NET, including patients with skeletal NET metastases (21).

### Impact of siNET BM on overall survival

Our findings also highlight the aggressive nature of siNET BM, with the majority developing within two years after diagnosis. Furthermore, our data indicate that BM significantly impacts OS, with a median OS of 127 months (10.6 years) for patients with BM compared to 170 months (14.2 years) for those without. This underscores the importance of early detection and personalized therapeutic approaches to enhance outcomes for patients with siNETs and BM.

In contrast, survival was considerably longer than reported in an analysis of the SEER registry data from the United States, where median OS for stage IV siNET patients was only eight years (22). The longer OS observed in our siNET cohort compared to previously published data may also reflect differences in healthcare systems, diagnostic tools, improvements in NET therapies and the benefits of structured, multidisciplinary management at a specialized ENETS center.

The distinctive characteristics of BM in siNET observed in our study prompt considerations about a potential unique biological variant compared to siNETs with distant metastases other than bone. The early manifestation of BM within two years of initial diagnosis suggests that these tumors may possess distinct molecular and genetic features involving specific pathways facilitating early dissemination to the bone. Notably, 41% of patients developed BM synchronously within the first six months after siNET diagnosis, further supporting the hypothesis that bone involvement may be an early and intrinsic feature of this siNET subset.

Almost all patients with BM also had visceral metastases (96.2%), most frequently involving the liver (90.5%), while exclusive bone disease was observed in only two patients. Patients with liver-only metastases did not show such poor survival, suggesting that the combination of liver and bone involvement confers a particularly unfavorable prognosis. However, given the very low number of patients with bone-only disease, the independent impact of BM cannot be definitively separated from that of simultaneous liver metastases.

### Study strengths and limitations

Compared to previous studies, our analysis offers several unique strengths. First, this is the largest single-center cohort to focus exclusively on histologically confirmed siNETs, minimizing the heterogeneity seen in prior multicenter or registry-based studies that often pooled different NET subtypes. Second, all patients underwent standardized imaging with 68Ga-DOTATOC-PET/CT, which is one of the most sensitive modalities for NET detection, allowing for early identification of bone metastases, including those visible only by tracer uptake. Third, the consistent, longitudinal follow-up in a certified ENETS Center using a “one-stop shop care” model ensured uniform management and high-quality data across the entire cohort. Importantly, our findings demonstrate a strong and previously underappreciated link between serotonin-producing siNETs and bone involvement. We also provide evidence that antiresorptive therapy may prevent skeletal-related events, supporting its early integration into treatment strategies. Finally, we identify BM as an independent prognostic factor for worse overall survival, highlighting its relevance for risk stratification and treatment planning.

At the same time, the retrospective design and the limited proportion of patients receiving antiresorptive therapy need to be considered when interpreting the impact on SREs and survival. Although SREs were completely absent under antiresorptives, treatment was not randomized, and confounding by patient selection cannot be excluded. Furthermore, molecular data were not available, precluding insights into potential biological drivers of early bone involvement.

## Conclusion

BM are more common in siNET than previously reported, most often with an osteoblastic morphology and an unfavorable prognosis. The impact on survival underscores the clinical significance of BM. Almost half of BM were initially visible only on functional imaging, highlighting the importance of systematic ^68Ga-DOTATOC PET/CT in staging and follow-up. Antiresorptive therapy was associated with complete prevention of SREs in this cohort, supporting its early use as part of multidisciplinary management of siNET patients with BM. The early manifestation of BM in siNETs challenges conventional views on the association between tumor burden and BM development suggesting a distinct subset. Prospective studies are required to clarify whether adapting current treatment algorithms for siNET including systematic skeletal assessment and routine antiresorptive therapy can improve NET management.

## Data Availability

The raw data supporting the conclusions of this article will be made available by the authors, without undue reservation upon reasonable request.
