# Supplementary figures and images for: High incidence and poor prognosis of bone metastases in functioning small intestinal neuroendocrine tumors
Source: Front Endocrinol (Lausanne). 2025 Oct 23;16:1680209. doi: 10.3389/fendo.2025.1680209 (PMC12588865; doi:10.3389/fendo.2025.1680209)

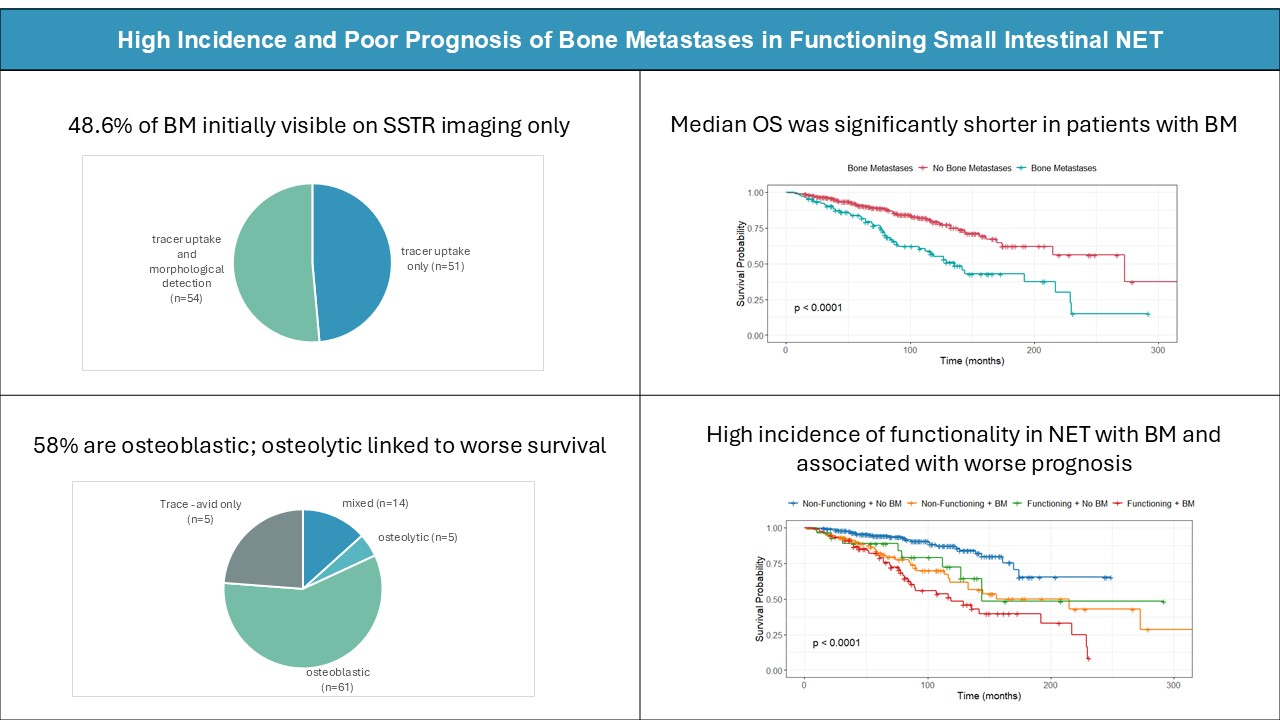

Supplement: Supplementary file 1 [file Image1.jpeg]
